# The Importance of Age Dependent Mortality and the Extrinsic Incubation Period in Models of Mosquito-Borne Disease Transmission and Control

**DOI:** 10.1371/journal.pone.0010165

**Published:** 2010-04-13

**Authors:** Steve E. Bellan

**Affiliations:** Department of Environmental Science, Policy and Management, University of California, Berkeley, California, United States of America; University of Leeds, United Kingdom

## Abstract

Nearly all mathematical models of vector-borne diseases have assumed that vectors die at constant rates. However, recent empirical research suggests that mosquito mortality rates are frequently age dependent. This work develops a simple mathematical model to assess how relaxing the classical assumption of constant mortality affects the predicted effectiveness of anti-vectorial interventions. The effectiveness of mosquito control when mosquitoes die at age dependent rates was also compared across different extrinsic incubation periods. Compared to a more realistic age dependent model, constant mortality models overestimated the sensitivity of disease transmission to interventions that reduce mosquito survival. Interventions that reduce mosquito survival were also found to be slightly less effective when implemented in systems with shorter EIPs. Future transmission models that examine anti-vectorial interventions should incorporate realistic age dependent mortality rates.

## Introduction

For many arboviral diseases, vector control remains the primary, and often only, tool for reducing disease incidence [1]. With climate change predicted to increase the transmission intensity and geographic spread of vector-borne diseases, preventative vector control becomes increasingly important [2]. The fundamental theory behind the management of vector-borne diseases arises from Macdonald's model of malaria formulated half a century ago [3]. His model provided many important intuitive explanations for why certain interventions are more effective than others at reducing transmission. As such, this model is still widely cited as theoretical support for adult mosquito control as the best management approach for vector-borne diseases [4], [5], [6]. Better theoretical insight into disease management can be attained, however, by thinking carefully through model assumptions for each transmission system of interest. In that way models can yield insight into how the effectiveness of control interventions varies between different vector-pathogen systems. This work examines how a simplifying mosquito mortality assumption affects the predicted effectiveness of adult mosquito control.

### The Ross-Macdonald model

Macdonald (1957) derived the following formula for the basic reproduction number (*R_0_*, defined as the number of secondary cases generated by an index case in an otherwise susceptible population) of vector-borne diseases by adding a latent period to Ross (1902)'s earlier model of malaria:
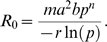
(1)


Because only adult female mosquitoes take bloodmeals, only they are modeled and hereafter all mosquitoes referred to are adult females unless otherwise specified. The parameters are defined in the following way: *m* is the ratio of mosquitoes to humans (or mosquito density if assuming constant human population), *a* is the biting rate (human bites per day per mosquito), *b* is the infectiousness of infected mosquitoes (proportion of bites that cause an infection), *p* is the daily survival rate of mosquitoes, *n* is the extrinsic incubation period (EIP; number of days between a mosquito's infection and when it can yield infectious bites), and *r* is the recovery rate of human infectious cases or the inverse of the duration of infectiousness [3]. Garrett-Jones (1964) emphasized the mosquito related components of *R_0_* in the vectorial capacity:
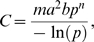
(2)


which avoids difficulties associated with estimating the duration of infectiousness in humans. *C* can be used as an index of transmission intensity, and is a theoretical property of a vector population defined as the number of secondary human infections that will be generated by a population of mosquitoes that is exposed to a single infectious human for one day. Both *R_0_* and *C* describe the potential for transmission in a disease-free system, and therefore extrapolation to endemic systems should be made carefully [7].

The Ross-Macdonald model yielded two strong qualitative conclusions that have greatly influenced vector-borne disease management [4]. Firstly, *C* scales with the square of the biting rate, *a^2^*. Thus, reductions in the bite rate (for example, via use of repellents or bednets for night-biting mosquitoes) should be moderately effective at reducing transmission. Secondly, *C* scales nonlinearly with the mosquito daily survival probability, as *p^n^/-ln(p)*, thus changes in the survival rate should be extremely effective at reducing transmission. This term, sometimes known as the longevity factor, is the product of the probability a mosquito survives through the EIP, *p^n^*, and its expected life expectancy after the EIP, *-ln*(*p*)^−1^ [4], [8]. In other words, in addition to reducing the number of mosquitoes in a system (e.g. reducing *m*), killing mosquitoes regulates transmission by reducing the probability mosquitoes survive to be infectious (especially when EIP is long) and reducing the average number of days they live (and bite) once infectious. With the long EIP of *Plasmodium* spp. (between 11–21 days [9]), *C* is very sensitive to reductions in the daily survival probability of *Anopheles* spp. mosquitoes (the primary vectors of malaria). Consequently, the majority of human malaria control programs in the last fifty years have focused on the adult rather than immature mosquitoes [4], [6], [10].

### Extensions of the Ross-Macdonald model to other vector-borne diseases

The Ross-Maconald model was formulated to describe transmission of *Plasmodium* spp. by primarily *Anopeheles* mosquitoes. Yet, this model and its extensions still have formed the backbone of models of many other vector-borne diseases including arboviruses [11], [12]. But because vector-pathogen dynamics differ substantially between systems, basic assumptions and the biological plausibility of interventions must be re-examined for each model. For instance, the EIP ranges for arboviruses [13], [14], [15] tend to be much shorter than the range for *Plasmodium* spp. [3]. Some arboviruses also have important multi-host dynamics (*i.e.* yellow fever virus and West Nile virus). Man-biting rates by *Aedes* spp. mosquitoes, important vectors of several arboviruses, may be much higher than those by *Anopheles* spp. because the former have a tendency to bite multiple hosts within a single gonotrophic cycle [16]. Further, some mosquito species can transmit arboviruses transmitted transovarially to their offspring, allowing for the pathogen to be maintained without transmission through other host species [17].

Behavioral differences between mosquito species may also make certain control measures effective for some vector-pathogen systems while ineffective for others. For instance, indoor residual spraying (IRS) and insecticide-treated bednets (ITN) can be very effective at reducing malaria transmission [18], [19], [20], [21] because many anopheline mosquitoes bite indoors at night and then rest on walls; yet IRS and ITN are less effective for control of *Ae. aegypti* [22], [23] (though with some exceptions [24]) which bite during the daytime and outdoors and therefore are less likely to be exposed to insecticide sprayed indoors [5]. Yet, in comparison to anopheline mosquitoes, *Ae. aegypti* thrive in urban environments and frequently breed in man-made containers [25] making certain community-based interventions more feasible [26]. Dynamical models of disease systems must consider these biological characteristics to yield productive insight.

### Controlling adult vs. immature mosquitoes

The Ross-Macdonald model does not include larval stages of mosquitoes and, therefore, no obvious parameter of *C* can be varied to realistically analyze the sensitivity of vectorial capacity to control of immature mosquitoes in this framework. The mosquito to human ratio (*m*) is an index of mosquito density and it may seem obvious that larval control affects transmission by reducing mosquito density. But the relationship between adult and immature mosquito density is not only unlikely to be linear but also variable across space, time, and species [27]. For some species, simple models of mosquito demography have been useful (*Culex tarsalis*; [28]), but for others more complicated population dynamics have required simulation models that, while more realistic, are too complex for tractable mathematical analyses (*Ae. aegypti*; [29], [30]). Therefore, there has been no strong theoretical conclusion about the utility of larval control, paralleling that of adult control. It has frequently been noted that appropriate implementation of larval control will require quantification of this relationship with field data [5], [27], [31] though some progress has been made [32].

Despite these theoretical deficiencies, control of *Ae. aegypti* for dengue management has focused on larval rather than adult control in recent years [5], due to the failure of outdoor ultra-low volume spraying campaigns [33]. Government implemented (vertical) larval-based control programs successfully eradicated *Ae. aegypti* from most of the Americas for yellow fever control in the 1950s and 1960s, but were not sustainable [33]. For that reason, the last twenty years of dengue management have focused on community-based (horizontal) larval control drawing on Gubler (1989)'s [33] claim that sustainable control of dengue can only happen at the community level.

The numerous community-based larval control programs in the last two decades have yielded varied degrees of success, as usually measured by reduced larval density in households [31]. However, the poorly understood associations between larval density and adult density, and between adult density and human disease burden for *Aedes* spp.-borne arboviral diseases make these entomological outcomes rather uninformative [5]. At least one study suggests a strong relationship between larval density and disease outcomes [32], but biases due to lack of randomization and appropriate controls in the study's design may also at least in part explain these results [31]. The utility of larval control thus deserves more study on both the applied and theoretical sides of research [5], [27], [31].

In response, a recent panel of vector control experts concluded that *Ae. aegypti* control for reducing dengue, yellow fever, and chikungunya transmission should focus on adult life stages because, “it has been known since the early 1900s that the most cost-effective means of preventing mosquito-borne disease is to target the adult vector, which transmits the pathogen,” referring to the Ross-Macdonald model, originally formulated to describe malaria transmission [5]. In this paper, it is examined whether conclusions regarding adult control derived from the Ross-Macdonald model can be unequivocally applied to *Ae. aegypti-*borne arboviral systems. In particular, incorporating more realistic age dependent mosquito mortality may alter the predicted effectiveness of anti-vectorial interventions and these changes may depend on a pathogen's EIP [34].

### Age dependent mortality

**Figure 1:**
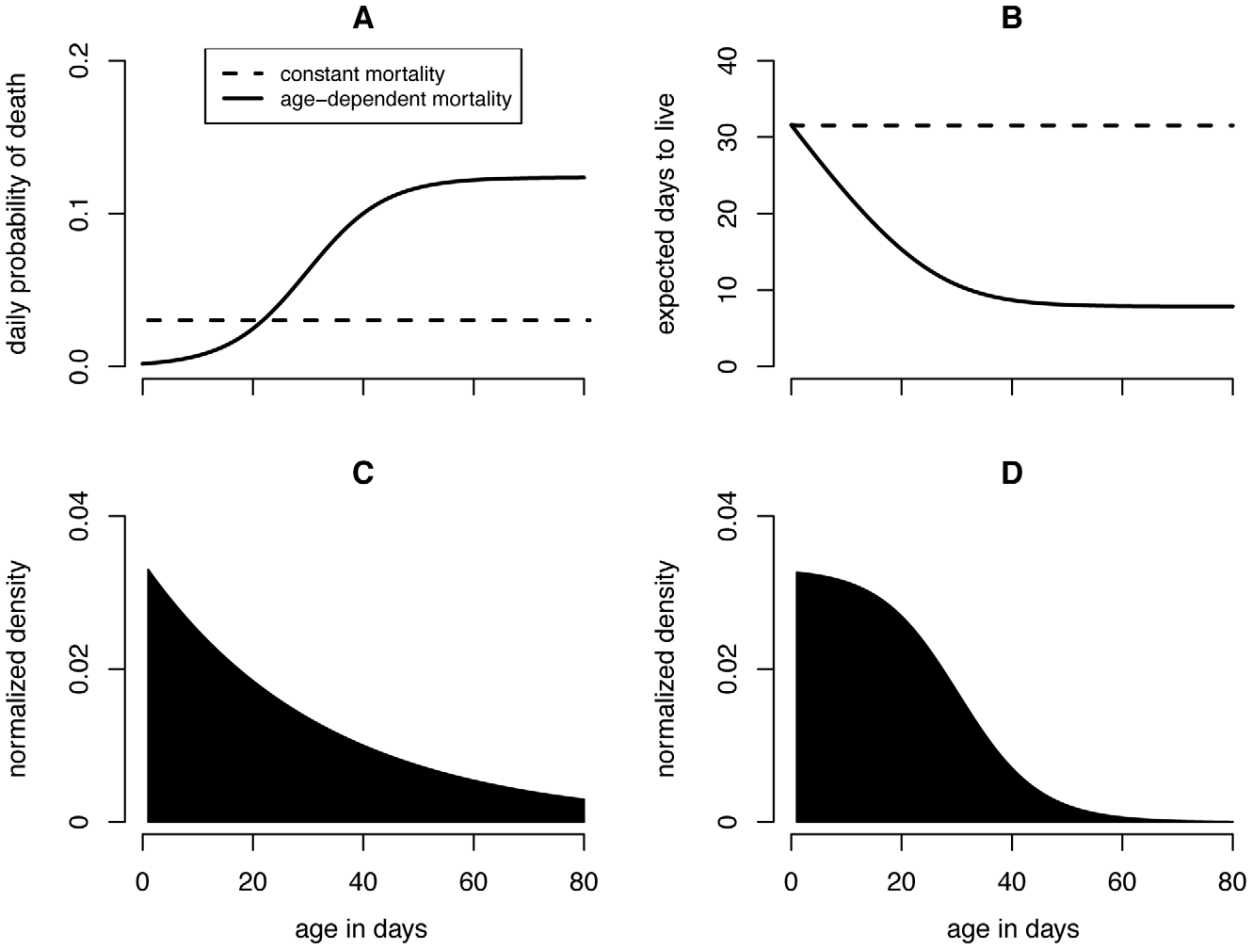
Mortality functions and mosquito demography. Panel (A) depicts the daily probability of death *p*(*x*) as a function of age for both the constant mortality model and the best fit logistic age dependent model to Styer et al. (2007)'s data. The resulting life-expectancy curves are displayed in (B); in both cases the life expectancy of a newly emerged adult mosquito is 32 days. In (C) and (D) the age distributions of a mosquito population experiencing constant and age dependent mortality, respectively, are shown. The number of mosquitoes has been normalized so that the total population density equals 1.

The notion that reducing the mosquito survival rate is particularly effective in regulating transmission stems from the *p^n^/-ln(p)* term in the vectorial capacity calculation. Due to the exponential nature of this term, mosquito survival and the EIP interact in a nonlinear manner to affect vectorial capacity. But mosquito senescence (*i.e.* the increase of the mortality hazard with age) further complicates this interaction [34]. Vector-borne disease models have with rare exception [35], [36], [37] assumed that vectors die at a constant rate based on the biological assumption that mosquitoes die of exogenous causes (swatting, predation, disease, weather) rather than endogenous causes (old age). By reanalyzing several mosquito age distribution data sets, Clements and Patterson (1981) [38] were among the first to challenge this assumption, demonstrating that several species appeared to exhibit age-dependent mortality patterns that are fitted well by a Gompertz mortality function. Styer et al. (2007) [34] investigated the relationship between mortality and age in a single age cohort of more than 100,000 *Ae. aegypti*, and found constant mortality rates to be unrealistic for this species, with the mortality hazard adequately fitted by either a logistic or a Gompertz function (Figure 1A). In a second smaller study, they confirmed age dependence of the mortality hazard for *Ae. aegypti* fed blood only, sugar only, or both blood and sugar [39].

Yet microcosm studies control many exogenous causes of death so that mosquitoes may have few remaining causes to die of other than old age. Therefore, it may be unsurprising that senescence occurs in such populations. In natural systems, endogenous causes of death will be less important because mosquitoes may rarely live long enough to die of old age. In such cases the mortality hazard should vary less with age. Since it is logistically unfeasible to directly observe mortality in wild mosquito populations, investigators have estimated age dependent survival rates either from age distribution snapshots of captured mosquitoes or from mark-recapture studies in which mosquitoes are aged. Yet both these approaches require age-grading wild mosquitoes. Such techniques exist but remain imperfect. To address this concern, Harrington et al. (2009) [40] simultaneously released multiple laboratory raised *Ae. aegypti* cohorts differing only by their age. By marking each age cohort with a different color dye and then recapturing mosquitoes, they were able to estimate survival rates as a function of age in a natural system. They also found survival to be age-dependent, with mortality rates an increasing function of age. Although more field studies with similar results will strengthen this conclusion, it seems clear that mosquitoes do senesce in some natural systems.

Different investigators have found different functional forms to provide the best fit to data (most frequently logistic, Gompertz, or Weibull functions). These functions describe mortality hazards that increase with age but at a decelerating rate. Some evidence indicates that the mortality hazard may actually begin decreasing at very old ages [34]. In a laboratory study of *Anopheles stephensi* mortality, Dawes et al. (2009) [41] found that a convex parabolic hazard function best fit their data. Thus, actual hazard functions are likely to depend on both the mosquito species and their environment. Regardless, it seems clear that the assumption of a constant mortality hazard does not hold in all cases.

While recent research has acknowledged the importance of age-dependent mortality in pathogen transmission [34], [38], [40], the effect of age dependent mortality on our understanding transmission dynamics has rarely been investigated. By creating an age dependent formulation of *C* using their best fit logistic model, Styer et al. (2007) [34] found that young *Ae. aegypti* adults hold the most transmission potential. This is because older mosquitoes have a lesser probability of surviving the EIP and a shorter life expectancy in an age dependent model. Consequently, age independent models overestimate the transmission potential of older mosquitoes and they overestimate *C*.

However, as pointed out by Dye (1992) [4], *C* calculations are intrinsically biased by the methodological difficulties associated with estimating its components. As the actual numerical values of *C* estimates will rarely be informative alone, studies should compare pre-control and post-control values of *C* to yield productive insight. Thus, the practical importance of age independent models overestimating *C* remains unclear. Dawes et al. (2009) [41] suggested that overestimation of *C* may lead constant mortality models to underestimate the effect of anti-vectorial interventions. Yet it is not immediately clear whether the assumption of a constant mortality hazard causes the effects of anti-vectorial interventions to be overestimated or underestimated without explicitly exploring *C*'s sensitivity to control under both mortality models.

Here, a model is developed to examine how age dependent mortality affects the ability of vector control measures to reduce *C*. Styer et al. (2007)'s [34] best fit logistic age dependent model is extended to incorporate two hypothetical classes of anti-vectorial interventions: reduction of adult mosquito survival or reduction of the adult mosquito recruitment rate. Styer et al. (2007)'s [34] best fit logistic hazard function is chosen because it is relevant to *Ae. aegypti* transmission, though other monotonically increasing functions are expected to give qualitatively similar results.

To de-emphasize actual numerical values of *C*, the following approach focuses on sensitivity analyses of *C* as a percentage of its value in the absence of control. In this way all results compare post-control scenarios to pre-control scenarios. Thus, emphasis is placed on whether percentage reductions in *C* for a given control parameter are greater or lesser in age dependent models compared to age independent models. Because mosquito mortality affects *C* in large part by reducing the probability a mosquito survives through an EIP, model output is explored for different EIPs. EIPs vary greatly within a single arboviral system as a function of vector species [15], temperature [42], and arboviral strain [15]. As an illustrative example EIP values are compared across the ranges for the dengue and chikungunya viruses whose EIPs in *Ae. aegypti* may range from as short as 2 days for the former [43] and as great as 12 days for the latter [14].

Specifically, this work aims to answer (1) how predicted mosquito control effectiveness is affected by replacing the classical assumption of a constant mortality hazard with an age dependent mortality hazard and (2) whether these results depend on the EIP. Interventions that increase adult mosquito mortality rates reduce the population density of the oldest mosquito age classes by the greatest proportion because older mosquitoes must survive through more days of mosquito control. Since old mosquito age classes already contribute less to vectorial capacity in age dependent models, it is hypothesized that in such models reducing mosquito survival will be less effective at controlling transmission, especially for diseases with shorter EIPs in which younger mosquitoes play an even greater role in transmission.

## Methods

The following model explores how age dependent mortality hazards affect the effectiveness of anti-vectorial interventions in reducing disease transmission. Model parameters are described in Table 1. Interventions aimed at the host population (such as vaccination or treatment) are not examined. Two hypothetical classes of vector control measures are considered: reduced survival of mosquitoes and reduction of mosquito recruitment. These interventions are theoretical and chosen because each intervention affects only one model parameter, facilitating a tractable analysis of how age dependent mortality may interact with interventions that affect mosquito demography. Actual control measures implemented in real systems affect many model parameters. For instance mosquito control not only increases mortality, but may also reduce the biting rate through the excito-repellent effect of many pesticides. Use of entomopathogenic fungal sprays to reduce survival may also lengthen the duration between two consecutive bloodmeals [44].

**Table 1:** Description of model parameters.

	Description	Value	Units	Ref
m	Ratio of mosq.^1^ to humans.	scaled^2^	mosq. per human	-
*a*	Biting rate per mosquito on humans.	scaled	bite × mosq^−1^ × day^-1^	-
*b*	Probability of transmission.	scaled	-	-
n	Extrinsic incubation period.	varied	days	[14], [15], [42], [43], [46]
*p*	Daily probability of death for adult female mosq. in constant mortality models.	0.030698	-	[34]
*x*	Mosquito age.	-	days	-
*P*(*x*)	Daily probability of death for adult female mosq. in age dependent mortality models.	function of *x*, α, β, γ	-	[34]
*Ω_x_*	Proportion of mosq. population of age x.	function of *p*(*x*) or *p*		
*e*(*x*)	Life expectancy of a mosquito of age *x*.	function of *p*(*x*) or *p*	days	[34]
α	Initial hazard^3^ for age-dependent models.	0.0018	death × mosq.^-1^ × day^-1^	[34]
β	Exponential increase in hazard with age for age dependent models.	0.1416	-	[34]
γ	Hazard deceleration for age-dependent models.	1.0730	-	[34]
σ	Mosq. age at first bloodmeal.	2	days	[34]
*r_A_*	Daily probability of mosq. death from control.	Varied	-	-
*r_L_*	Proportional reduction in mosq. recruitment from control.	Varied	-	-
*i, j*, *k*	Indices for calculating mosq. survival parameters in discrete time steps.	-	days	-

1 In this table the abbreviation mosq. indicates adult female mosquitoes.

2 Scaled indicates that this parameter has been normalized to one in the model because the sensitivity analyses are assumed not to interact with such parameters.

3 Hazard refers to the instantaneous rate of mortality.

Reduction of mosquito recruitment reflects real world interventions that focus on immature mosquito control. Such interventions do not only reduce recruitment directly, however, they also reduce larval habitat (*i.e.* source reduction) such that mosquitoes have fewer ideal places to deposit eggs. Larval density also affects larval mortality [25] and the size of emerging mosquitoes [45] which may subsequently affect biting rate and survival. Thus other important feedbacks between larval and adult population dynamics may occur. As such, the relationship between larval survival and recruitment are complex and require more theoretical and empirical work for clarification. For these reasons, recruitment is considered the control parameter of interest rather than immature mosquito survival or carrying capacity. Thus, control of recruitment is only modeled because, in contrast to reducing mosquito survival, it does not affect adult mosquito demography and therefore facilitates a comparison between these two hypothetical types of interventions.

Because of the above assumptions regarding anti-vectorial interventions, the biting rate and infection probability are considered constant scalars that do not interact with control parameters. These scalars are consequently removed from the model yielding the scaled vectorial capacity:
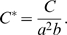
(3)


In the Ross-Macdonald model, C* becomes the product of the longevity factor and the density of mosquitoes:
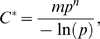
(4)


*C* is defined as the number of secondary human cases infected by a mosquito population exposed to an infectious human for one day. Therefore, *C*'s units are humans infected per day of human infectiousness. Dividing by *b* (a probability) does not change the units of C*. Rather, it equates to assuming that all mosquitoes that bite humans become infected. Dividing by the biting rate squared, however, does yield different units for C* than for *C*. The longevity factor (*p^n^*/-ln(*p*)) is simply the expected number of infectious biting days produced by a single mosquito if infected. Thus, C* is equivalent to the expected number of infectious biting days delivered by a mosquito population were all mosquitoes to be infected. Thus, the units of C* are infectious biting days given infection of the entire mosquito population. Of course, the entire mosquito population (or even a large proportion thereof) will almost never become infected in a real system. Nevertheless, C* can be used as a demographic index of a mosquito population's transmission potential. Mosquito populations with greater survival probabilities (*p*) hold the potential to deliver more infectious bites, if infected, both because they are more likely to survive the EIP (*n*) to become infectious and because they will live for longer once infectious. Longer EIPs lead to fewer infectious biting days with all else held constant.

Styer et al. (2007)'s [34] discrete age dependent model requires a slightly more complicated formulation, which reduces to the above equation when *p*(*x*) = *p* and *σ* = 1:
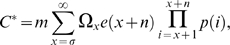
(5)


where Ω*_x_* is the fraction of mosquitoes that are of age *x*, *σ* is the age at when mosquitoes begin biting, *p*(*x*) is the daily survival probability of a mosquito of age *x*, and *e*(*x*+*n*) is defined as the life expectancy of a mosquito of age *x*+*n*. The value *e*(*x*+*n*) can be more intuitively thought of as the expected number of infectious biting days lived by a mosquito infected at age *x* if it survives the EIP. Taking the grand product of *p*(*i*) over the EIP yields the probability a mosquito survives through the EIP given it became infected at age *x*.

The best fit model describing the instantaneous mortality rate as a function of age, *µ*(*x*), from Styer et al. (2007)'s [34] empirical study of *Ae. aegypti* mortality was a logistic model (Figure 1A; parameters defined in Table 1):
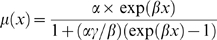
(6)


which can be converted into a daily probability of death for a discrete daily demographic model in the following way:
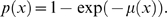
(7)


The expected number of infectious days is calculated using the usual formula for life expectancies (*i.e.* by summing under the discretized survival curve from age *x*+*n* forward; Figure 1B):
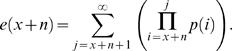
(8)


This equation can be understood by noting that summing over *j* equates to summing over the potential ages a mosquito that survived through age *x*+*n* could die, and that the addends to be summed are the proportion of mosquitoes who live to age *j* given they lived to age *x*+*n*.

The effect of reducing mosquito survival on C* is easily analyzed by replacing the intrinsic daily survival probability with the daily survival probability with mosquito control,
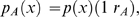
(9)


and conducting a sensitivity analysis to the control parameter, *r_A_*, which is defined as the daily probability of death due to control. It is emphasized here that this is a population model in which death probabilities are averaged across individuals and vector control measures are averaged across time. Thus, while insecticides may achieve nearly 100% death rates in mosquitoes where and when they are sprayed, spatial and temporal variability in spraying yield a much smaller probability of death from control for each mosquito for each day.

Increases in *r_A_* decrease not only the survival probabilities of mosquitoes, but also their density, *m*. This can be incorporated into the model by taking 
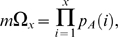
(10)


so that *m* is normalized to 1, and Ω*_x_* is simply the proportion of mosquitoes that survive to age *x*. Note that the proportional reduction in the density of a mosquito age class is 1–(1–*r_A_*)*^x^*, which increases as a function of mosquito age, *x*, because older mosquitoes have had to survive through more days of control. Substituting this equation into equation [5] yields
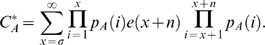
(11)


Equation 11 can be restructured in the following way
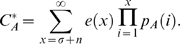
(12)


Note that, as in equation [4], scaled vectorial capacity can now be expressed as the proportion of mosquitoes who live to *σ*+*n* days or older, multiplied by their life expectancy at that age, and summed across age classes. This is equivalent to the expected number of infectious biting days a mosquito in this population would yield if infected. As noted above, incorporation of larval control is much more nuanced. Because this relationship is not well understood this model, for simplicity and to draw a contrast with reductions in mosquito survival, only examines proportional reductions in recruitment, *r_L_*:
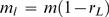
(13)


Reductions in recruitment therefore reduce the number of mosquitoes in all age classes by an equal proportion:
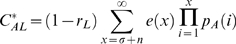
(14)


Adding these control measures yields the following constant mortality model:
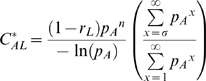
(15)


where the latter term is the proportion of the mosquito population older than than *σ* days.

Using these equations a sensitivity analysis to *r_A_* and *r_L_* was conducted for both mortality models and for the EIPs ranging from 2 to 12 days, reflective of the shortest duration for the chikungunya virus [43] and the longest duration for the dengue viruses [42]. Importantly, the average mosquito lifespan does not vary between constant and age dependent models. In both cases, mosquito life expectancy at emergence is 32 days old in the absence of control [34]. These models differed only by their functional specifications of the mosquito death rate and, consequently, the response of their population sizes and age distributions to control. These, in turn, predict different expected number of days infected mosquitoes will live (and bite), thereby changing their predicted vectorial capacity. All simulations and figures were produced in the statistical package ‘R’ (code provided in File S1).

## Results

**Figure 2:**
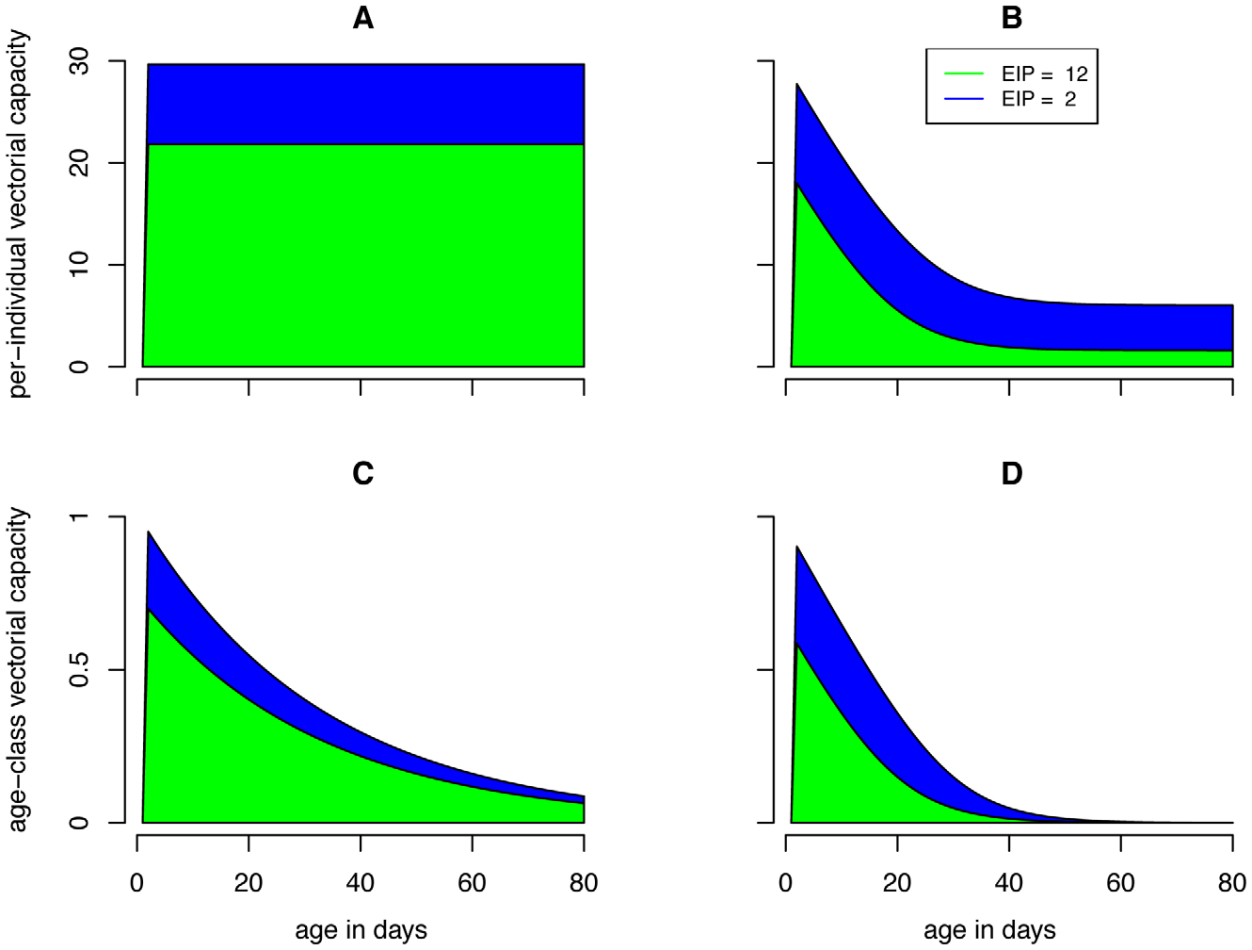
Vectorial capacity contribution by age and age class. Panels (A) and (B) show the individual-level contribution to scaled vectorial capacity (C*) by age; and panels (C) and (D) show the contribution of each age class to C*. The left panels assume constant mortality rates while the right panels assume age dependent mortality rates. Pathogens with shorter extrinsic incubation periods (EIP) allow mosquitoes to transmit sooner after they become infected. For that reason, mosquitoes of all ages contribute more to C*. The blue region shows the extra C* contributed for an EIP of 2 in addition to that for an EIP of 12 given all else held constant. The sensitivity of C* to the EIP is more dramatic in the age dependent model (D) compared to the constant model (C).

Relaxing the assumption of constant mortality yields mosquito populations that are much more skewed towards younger age classes (Figure 1C–D). Because C* can be formulated as in Equation 12, scaled vectorial capacity can be seen as the sum across ages of mosquitoes that are old enough to be infectious (*i.e. n*+*σ* days old or older) of the product of mosquito density and life expectancy. Figure 2 shows how the individual-level and age class level contribution to C* compares between mortality assumptions. Allowing for age dependence reduced the individual-level contribution of older mosquitoes as well as the number of old mosquitoes in a population, and thus greatly decreased the importance of older mosquitoes in disease transmission. As a result, the proportional increase in C* due to shortening the EIP from 12 to 2, which allows younger mosquitoes to be infectious, is much greater in the age dependent model (Figure 2C–D).

**Figure 3:**
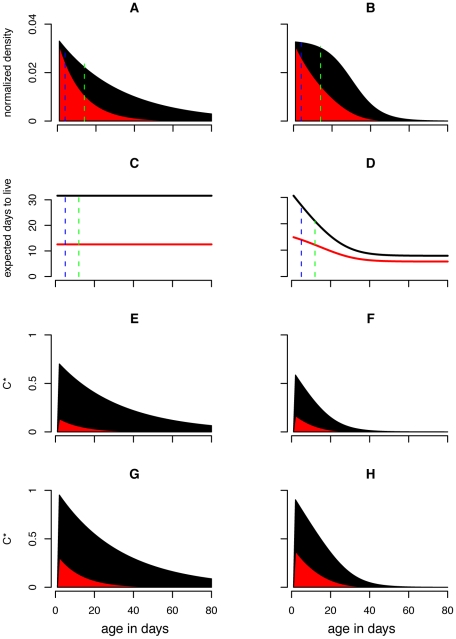
Effects of reduced mosquito survival on demography and vectorial capacity. The above figure displays the effects of reducing mosquito survival (*r_A_* = 0.05, red) compared to a baseline scenario (*r_A_* = 0, black) on the mosquito population distribution (A,B), the life expectancy function (C,D) and the C* contributions by age class for extrinsic incubation periods of 12 days (E,F) and 2 days (G,H). The left column of panels (A,C,E,G) displays results from the constant mortality model while the right column of panels (B,D, F, H) show results from the age dependent mortality model. The proportional reduction in C* due to reducing survival is greater for the constant model than for the age dependent model, and greater for the longer EIP. The green and blue dashed lines in the first two rows indicate the youngest age at which mosquitoes can be infectious when the EIP is 2 and 12 days, respectively. Because C* can be calculated by summing across ages old enough to be infectious the product of mosquito density and life expectancy (Equation 12), these dashed lines can be used to visually inspect how the first two rows of panels yield the second two. The region between the dashed lines in panels A and B consist of the mosquitoes who are too young to be infectious for an EIP of 12 days but are old enough to be infectious for an EIP of 2 days. While the proportional reduction of these age classes due to reducing mosquito survival is relatively small compared to older mosquitoes, the proportional reduction in life expectancy in these age classes is quite large. These effects almost balance each other out such that interventions that reduce mosquito survival are only slightly less efficient for shorter EIPs (see Figure 5).

**Figure 4:**
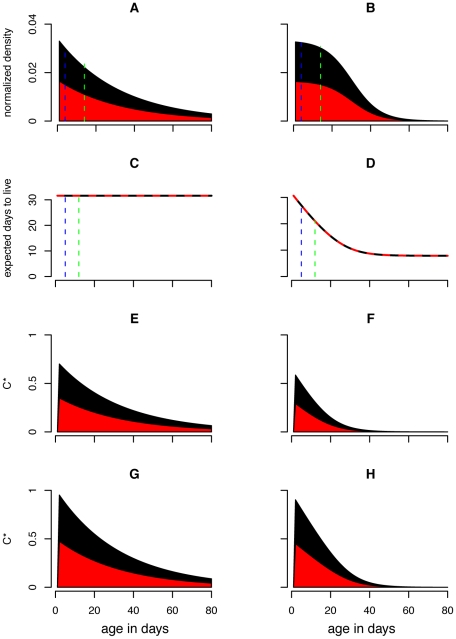
Effects of reduced recruitment on demography and vectorial capacity. The above figure displays the effects of reduced recruitment (*r_L_* = 0.5, red) compared to a baseline scenario (*r_L_* = 0, black) on the mosquito population distribution (A,B), the life expectancy function (C,D) and the scaled-vectorial-capacity-contributions by age class for extrinsic incubation periods (EIP) of 12 days (E,F) and 2 days (G,H). The left column of panels (A,C,E,G) displays results from the constant mortality model while the right column of panels (B,D, F, H) show results from the age dependent mortality model. The green and blue dashed lines in the first two rows indicate EIPs of 12 and 2 days, respectively. Because C* can be calculated by summing across ages old enough to be infectious the product mosquito density and life expectancy (Equation 12), these dashed lines can be used to visually inspect how the first two rows of panels yield the second two panels. Note that, unlike reducing mosquito survival (Figure 3), reducing mosquito recruitment by half reduces scaled vectorial capacity (C*) by half in regardless of the hazard model or EIP.

**Figure 5:**
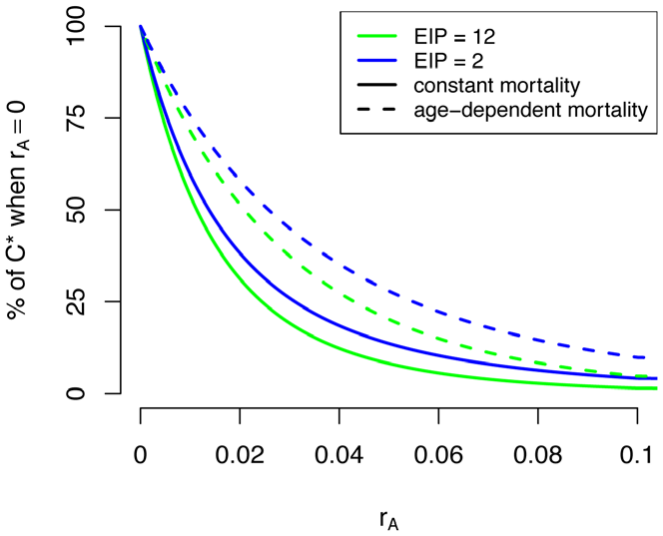
Sensitivity of vectorial capacity to mosquito survival. The above figure shows a sensitivity analysis of scaled vectorial capacity (C*), as a percentage of its baseline value (*i.e.* when *r_A_* = 0), to increasing levels of mosquito mortality from anti-vectorial interventions (*r_A_*>0). Note that the age dependent models are less sensitive to reductions in survival in that they require greater *r_A_* to achieve the same percentage reduction in C*. Additionally, shorter extrinsic incubation periods (EIP) make C* in age dependent models slightly less sensitive to *r_A_*.

**Figure 6:**
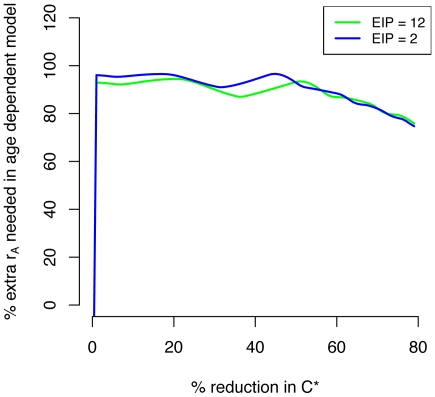
Constant hazard assumptions cause models to overestimate effectiveness of reduced mosquito survival in reducing transmission. The percent increase in the daily probability of death due to intervention (*r_A_*) required to reach a threshold percentage reduction in scaled vectorial capacity (C*) in an age dependent mortality model compared to a constant mortality model is plotted as a function of the threshold reduction desired. For most meaningful percentage reductions in C*, the age dependent model predicts that *r_A_* will need to be about twice as great compared to the constant model. The constant model overestimates control efficiency by nearly the same amount regardless of the extrinsic incubation period (EIP).

While control of recruitment and survival both reduce the mosquito population size, these reductions partitioned across age classes differently. Reductions in survival led to disproportionately large reductions in the population size of older age classes (Figure 3A–B). Control of survival also affects mosquito life expectancy and therefore its effects on C* were more nuanced. Because the effectiveness of reducing recruitment was unaffected by mortality assumptions it decreased C* by the same proportion (*r_L_*) in all cases (Figure 4). Under the constant mortality assumption, control of survival reduces the life expectancy of all mosquitoes by a large amount (Figure 3D); when age dependence is allowed, the reduction in life expectancy was less dramatic and skewed towards younger age classes (Figure 3E). So while reducing survival dramatically reduced the C* contribution of older mosquitoes in a constant mortality model, this effect is less important in age dependent models (compare Figure 3E with 3F and 3G with 3H). Because of these dynamics, the constant mortality model overestimates the effectiveness of reducing survival in controlling transmission (Figure 5). To achieve the same proportional reductions in C*, age dependent models required about twice as great of a reduction in survival compared to the constant models (Figure 6).

Mosquitoes older than *n*+*σ* days can contribute to transmission. Thus, for shorter EIPs (*n*), younger mosquitoes are able to contribute to transmission. Because interventions which affect survival minimally reduce the population size of young mosquito age classes (Figure 3A–B), this form of control was slightly less effective for shorter EIPs, in which younger mosquitoes play a greater role in transmission. This can be seen by the slightly slower rate of exponential decay for the C* curves corresponding to shorter EIPs in Figure 5.

## Discussion

These results demonstrate how assuming a constant mortality hazard can bias mathematical models of vector-borne diseases and their control when actual mortality hazards vary with age. The standard constant mortality assumption leads to overestimates of C* as found by Styer et al. (2007) (Figure 2) [34]. This occurred for two reasons. First, all but the youngest of mosquitoes had a greater probability of surviving through the EIP in the constant mortality model and therefore contributed more to C*. Second and more importantly, the expected number of days a mosquito will live once it has survived the EIP (and subsequently the expected number of bites an infectious mosquito delivers before it dies) differed between models. In the constant mortality model this value was always 32 days regardless of age. In the more realistic age dependent model, this value was 32 days for a newly emerged mosquito but decreased with increasing age. Consequently, the vectorial capacity contribution on a per mosquito basis remained constant in the constant model but decreased with increasing age in the age dependent model (Figure 2A–B).

It is more insightful, however, to explore the sensitivity of C* on a proportional rather than absolute scale because C* is a theoretical quantity and its absolute value is difficult to quantify. On this relative scale, the sensitivity of C* to reductions in the daily probability of death due to control (*r_A_*) was found to be greater in the constant mortality model than in the age dependent model (Figure 5). This is because the densities of older mosquito age classes are more sensitive to increases in *r_A_* than those of younger age classes (Figure 3). In age dependent models older mosquitoes already contribute much less C* than in constant mortality models. Therefore, the reduction in C* caused by their removal is less and interventions that reduce survival rates are less effective when natural mortality is age dependent. These results then suggest that the common assumption of a constant mortality hazard has led most vector-borne disease models to overestimate the efficiency of mosquito control. Models using an empirically derived age dependent hazard predicted that about double the level of control will be needed to achieve the same proportional reduction in C* as compared to constant hazard models (Figure 6). Consequently, these results contradict Dawes et al. (2009)'s [41] suggestion that constant mortality models may have underestimated the effectiveness of anti-vectorial measures.

Younger mosquitoes should contribute relatively more towards disease transmission when EIPs are shorter because mosquitoes can be infectious at a younger age. Again, increases in *r_A_* reduce the population density of older age classes by greater proportions than younger age classes. Thus, it was hypothesized that shorter EIPs would lead C* to be less sensitive to interventions that affect survival. While this was the case, the effect was less than expected (Figure 5). In the age dependent model, increasing *r_A_* reduced the life expectancy of younger mosquitoes much more than that of older mosquitoes (Figure 3D). Thus the greater sensitivity of life expectancy to *r_A_* for younger mosquitoes partly balances out the greater sensitivity of older mosquito age class densities to *r_A_*. Consequently, the age dependent model suggests that interventions that reduce survival are only slightly less efficient for shorter EIPs (with all else held constant).

Because control of recruitment (*r_L_*) alone does not interact with survival or life expectancy functions (Figure 4), its effectiveness is the same in both the constant and age dependent models. But without a better data-driven understanding of the relationship between larval control and recruitment caution must be used when drawing practical implications on the utility of larval control from mathematical models. Larval density affects larval mortality, rate of larval maturation, and size of emerging mosquitoes [25], [45]. Density dependent dynamics in larval populations may therefore greatly affect the utility of larval control and deserve further attention in the age dependent paradigm.

Because a static model was employed, no dynamic relationship existed between adult and immature mosquitoes. This means that, in this model, killing mosquitoes or reducing recruitment did not lead to less reproduction because reproduction was not formally specified. While unrealistic, this simplification emphasized the effects of *r_A_* on mosquito survival, life expectancy and age distribution. If a population dynamics model were used, these fundamental results would become clouded by arbitrary specifications of reproduction and density-dependence in the larval stages. However, in reality killing mosquitoes will also affect recruitment because fewer mosquitoes will be there to reproduce. At the same time, larval control will not only reduce recruitment directly but also indirectly by also reducing the number of mosquitoes that are reproducing.

The above model is very simple and therefore has several limitations. The age dependent mortality model was taken from Styer et al. (2007)'s [34] microcosm study of mosquito mortality rates. The classical assumption of constant mortality is stemmed from the intuition that mosquitoes die, not of old age, but of environmental causes that are not age related. It is possible that the confined nature of this empirical study limited these environmental causes of death and therefore yielded a greater proportion of age related deaths than would exist in a natural population. However, Harrington et al. (2009) [40] provide evidence for age dependent mortality in a natural population *Ae. aegypti* in Thailand. Further field studies of mosquito age distributions are necessary to determine the many factors that determine the extent to the mortality hazards of wild populations are age dependent. For example, Styer et al. (2007) [39] found qualitative differences between hazard rates for mosquitoes offered blood on a daily basis compared to those offered blood every other day. In general, populations living in less ideal environmental conditions, either due to high levels of predation or poor climactic conditions (both of which are unlikely to cause mortality in an age dependent fashion), should exhibit hazards that change less with age.

Qualitatively different hazard functions may yield different results. For example, Dawes et al. (2009) [41] studied the demography of a laboratory raised cohort of *An. stephensi* and found them to experience an initially high mortality hazard (presumed to be associated with taking their first bloodmeal) before decreasing to a minimum and then increasing again with age. Initially high hazards would reduce the importance of the youngest mosquito age classes, leading those of intermediate age to hold the greatest transmission potential because of their greater life expectancy. When the youngest and oldest age classes experience high hazards, biases due to assuming constant mortality should partly balance each other out. Thus biases due to assuming a constant hazard should be less for mosquito populations that, in reality, experience a convex parabolic hazard (as in Dawes et al. (2009) [41]) vs. a monotonically increasing hazard.

Another important point is that this model assumes that all mosquitoes are currently uninfected and only considers their transmission potential if exposed to infected humans. While this is applicable before and at the beginning of outbreaks, reducing transmission in the midst of outbreaks or in endemic areas should consider a partially infected mosquito population. Reducing mosquito survival will undoubtedly be more important for reducing human incidence when some of the adults to be killed are already infectious. However, this will depend on the prevalence of infectiousness amongst mosquitoes at any given time and should be explored using a stochastic model when very few mosquitoes are infected. For simplicity, the model also assumed that the mosquito population was at equilibrium and that mosquito control was applied homogenously across individuals and constantly over time. Different types of mosquito control are applied on different schedules and with different spatial regimens, which may affect transmission dynamics substantially. Seasonal and spatial dynamics and their interaction with different forms of mosquito control deserve further work.

There are, of course, other important means of managing arboviral diseases that are ignored in this model. These include those that reduce the man-biting rate (repellents, insecticide treated nets, indoor residual spraying), those that remove larval habitat (source reduction), and those that prevent infection in or shorten the infectious period of human hosts (*i.e.* vaccination in the case of yellow fever). These methods were not considered because the purpose of the model was to highlight how age dependent mortality interacts with mosquito control.

### Conclusion

Using a simple model of vector-borne disease transmission, it was demonstrated that the classical assumption that mosquitoes die at a constant (age independent) mortality rate has led most transmission models to overestimate the effectiveness of interventions which reduce the mosquito survival rate. Future models of vector-borne disease should incorporate age dependent hazards if they consider sensitivity of disease transmission to mosquito control or fit models of control to data. Reductions in mosquito survival still produce an approximately exponential decline in transmission intensity and therefore, when logistically feasible, should remain an important tool for vector-borne disease management. In systems with shorter EIPs, interventions that reduce mosquito survival are less effective in limiting transmission. But the reduction is very slight and should be considered negligible when making decisions regarding disease management.

## Supporting Information

File S1 This ‘R’ script codes the model in its entirety as well as produces all figures as they are shown in the manuscript.
(0.06 MB TXT)
